# Synthetic Content Validation of Pediatric Trust Instruments Using Persona-Driven Large Language Models: Development and Evaluation Study

**DOI:** 10.2196/96901

**Published:** 2026-10-13

**Authors:** Ella Boone, Katya Loban, Carmel Daskalo, Suganthi Rajasegaran, Elena Guadagno, Jeannie Haggerty, Sylvie Lambert, Dan Poenaru

**Affiliations:** 1 Department of Pediatric Surgery, Faculty of Medicine and Health Sciences, McGill University, 3D-5252 Blvd de Maisonneuve O, Montreal, QC, H4A 3S9, Canada, 1 2898342690; 2 Harvey E. Beardmore Division of Pediatric Surgery, Montreal Children's Hospital, McGill University Health Centre (MUHC), Montreal, QC, Canada; 3 Department of Family Medicine, Faculty of Medicine and Health Sciences, McGill University, Montreal, QC, Canada; 4 Ingram School of Nursing, Faculty of Medicine and Health Sciences, McGill University, Montreal, QC, Canada

**Keywords:** generative AI, large language model, pediatrics, psychometrics, computer reasoning, medical informatics

## Abstract

**Background:**

Large language models (LLMs) could assist in streamlining health care instrument validation. This is particularly relevant as traditional instrument development is time-intensive and resource-intensive. Currently, there is a significant gap in specifically developed and validated trust instruments for pediatric emergency and surgical contexts. Such tools are key because trust is foundational to the patient family–physician relationship and is associated with improved care-seeking and treatment adherence.

**Objective:**

This study aimed (1) to develop new trust instruments and conduct their preliminary content validation confirming item relevance through a Synthetic Instrument Validation approach integrating human and LLM capabilities and (2) to evaluate appropriate use cases for LLMs in psychometric assessment.

**Methods:**

Two trust instruments were developed (patient family and physician versions). In phase 1, instruments underwent a 2-stage content validation process using parallel synthetic and human expert panels (5 patient family members, 3 physician synthetic experts, and 7 unique human experts). Synthetic panels consisted of 3 persona-prompted LLMs (Claude Sonnet 4, GPT-5, and Grok 4), with human panels serving as comparators. The scale–content validity index (S-CVI) threshold was set at ≥0.80, percent agreement on item relevancy was used to compare panels. In phase 2, LLM performance in quantitative research tasks was evaluated. The patient family instrument underwent Flesch-Kincaid assessment; both instruments underwent cosine similarity analyses using algorithmic and LLM-based methods.

**Results:**

In phase 1, percent agreement between panels for the patient family and physician instruments was 90.9% and 88.9% for dimensional validation and 90.9% and 100% for contextual validation. Fleiss κ for within-panel agreement ranged from poor to almost perfect; 0.20 (95% CI -0.23 to 0.63) to 1.00 (95% CI 1.00-1.00). All instrument subsections met or exceeded S-CVI thresholds across both rounds and panels. Phase 2 Flesch-Kincaid metrics converged across all 3 methods to target benchmark (mean Grade Level 8.1, SD 1.1; mean Reading Ease 60.1, SD 5.6), while reference readability was near target, showing convergence across implementations of the same formula. In contrast, cosine similarity analyses revealed significant LLM performance limitations; cosine similarity analyses were, therefore, abandoned in favor of the algorithmic method alone, which achieved a maximum cosine similarity of 0.83, indicating acceptable item distinctiveness overall.

**Conclusions:**

Persona-prompted LLMs effectively assisted preliminary content validation and may help reduce timelines from months to weeks, but showed limitations in quantitative computations. This suggests that, in this study, persona-prompted LLMs performed better in subjective rating tasks than in deterministic cosine similarity computations. These findings help illustrate task-dependent boundaries for LLM integration in psychometric research, necessitating selective human-LLM collaboration. While these instruments achieved preliminary content validity, they require full psychometric testing before they can be considered validated for clinical use. Synthetic Instrument Validation offers a feasible approach to preliminary health care instrument development while preserving key elements of content validation.

## Introduction

Health care measurement instruments are valuable tools for assessing aspects of clinical care and informing evidence-based improvements [1]. These instruments take multiple forms including questionnaires, scales, and index measures, and can assess attitudinal or latent constructs in patient care that are not otherwise easily measurable. Careful construction is, therefore, critical to ensure the findings accurately reflect the concept being measured [2,3].

Designing and validating new health care instruments, particularly attitudinal ones, is a complex, time-intensive, and resource-intensive process that can take months to years. Challenges include recruiting experts, particularly in specialized fields, a lack of a systematic validation framework, and difficulty generalizing to diverse populations [1,4]. As a result, researchers may resort to using existing instruments not designed for their particular setting or construct. One such example is the Pediatric Trust in Physician Scale (PEDI-TiPS), an instrument originally developed for adult primary care settings that was subsequently adapted for use in pediatric ambulatory care [5]. On the other hand, newly developed instruments may never progress past initial development, remaining untested. In either case, validation barriers prevent these instruments from meaningfully impacting patient care.

AI is rapidly transforming health care research, offering new capabilities across both quantitative and qualitative methodologies [6]. Synthetic AI data have become increasingly acceptable for text classification, data-driven decision-making, and predictive analytics [7,8]. They have been used to augment sparse datasets in disease modeling and to simulate how policy changes could impact patient populations [7,9]. Yet their application to instrument validation remains largely unexplored, representing an opportunity to address longstanding barriers that can slow or halt the process. Large language models (LLMs) in particular offer a promising avenue, with emerging evidence supporting their capacity to simulate expert judgment and function as quantitative coresearchers alongside human experts and computational benchmarks [10-13].

To test the synthetic approach, our team identified a gap in the literature regarding how trust is built between patients’ families and physicians in pediatric surgical and emergency settings [14]. Trust in these brief, inherently stressful, and high-stakes clinical settings is an ideal candidate for measurement, as it requires a distinct conceptualization from the well-studied primary care context, given the unique nature of the tripartite doctor-patient relationship [15-18]. Research in primary care has nonetheless demonstrated the broader importance of trust in health care, showing that greater trust is associated with higher care satisfaction, increased care-seeking, improved treatment adherence, and stronger continuity of care [19-23].

Some studies have applied modified scales originally developed for adult primary care settings to pediatric emergency contexts; however, none have examined the nuances of trust-building within these unique settings [5,14,15]. To operationalize trust for this study, we define it as the patient’s acceptance of a vulnerable situation and the degree of certainty that the physician will act in their best interest [14,24]. Accordingly, this study aims to develop instruments to measure trust and its key components in pediatric emergency and surgical care settings, from both patient family and physician perspectives.

To address the barriers inherent in instrument construction, we propose a framework that we term Synthetic Instrument Validation (SIV), which conceptualizes LLMs as complementary collaborators rather than replacements for human expertise or existing standard methods. This framework evaluates the appropriateness of LLM-assisted approaches across both quantitative and qualitative tasks that comprise instrument validation. Our study has two objectives: (1) to conduct preliminary content validation of new trust instruments (ensuring that item content is relevant and comprehensive enough to progress to subsequent psychometric validation) by integrating parallel synthetic and human expert panels to reduce feasibility barriers, and (2) to evaluate, as a subsequent step before progression to cognitive interviewing and piloting, whether LLMs can reliably function as quantitative coresearchers in tasks needed to confirm instrument readability and item nonredundancy. Together, these objectives help clarify where LLM-assisted approaches may be most appropriately applied throughout the instrument validation pipeline. The SIV framework emphasizes the synergistic relationship between human judgment and LLM capabilities [25]. We anticipate that this approach will reduce time and recruitment constraints in preliminary instrument validation, with the end goal of facilitating instrument development to allow for data-informed improvements in patient care in pediatrics and beyond.

## Methods

### Phase 1: Instrument Development and Validation

We developed and conducted preliminary validation of 2 instruments to assess trust levels and their constituent components, measured across 4 dimensions: competence, caring, communication, and dependability (Figure 1) [14]. One instrument assessed patient families’ experiences and perceptions of trust building in these settings, while the physician version evaluated perceived trust levels and strategies to enhance them.

**Figure 1.**
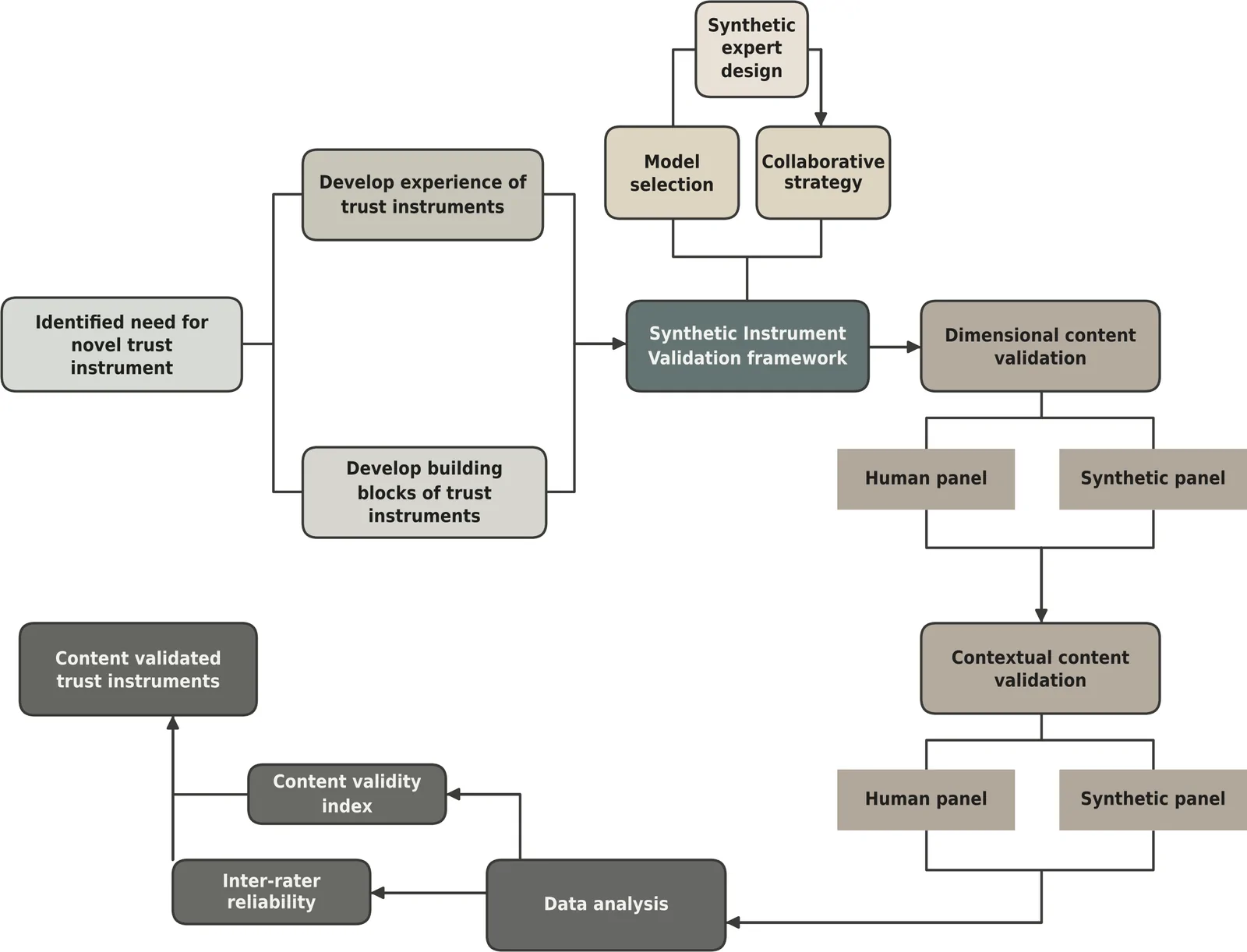
. Overview of methods for trust instrument development and content validation.

#### Experience-Based Trust (Subsection 1)

Based on a previous systematic literature review performed by our team [14], the Wake Forest Trust in Physician Scale (WFTPS) was selected as the starting point for item generation, with permission granted by the original authors for modifications [26]. This 11-item scale assesses fidelity, competence, honesty, confidentiality, and global trust, and demonstrates strong psychometric properties (Cronbach *α*=.93). Its prior use across diverse populations supported adaptation to the pediatric emergency and surgical contexts, as well as to physician self-reported contexts [27,28].

The patient family instrument incorporated elements of the WFTPS alongside newly developed relevant elements; this subsection was named the Trust-Experience–Patient-Family (TRUST-E-F). Review panel meetings comprising a pediatric general surgeon, a general surgery resident, 2 pediatric emergency physicians, and a quantitative researcher organized items into the 4 trust dimensions.

The corresponding physician subsection (Trust-Perception–Physician [TRUST-P-MD]) mirrored the TRUST-E-F items but was reframed from the physician perspective. Equivalent review meetings classified items into the 4 trust dimensions.

#### Building Block of Trust (Subsection 2)

The patient family (Trust-Building Blocks–Patient-Family [TRUST-BB-F]) and physician (Trust-Building Blocks–Physician [TRUST-BB-MD]) subsections were designed to capture factors potentially important to trust building, intended for concurrent completion alongside subsection 1. The same review panel approach was applied to align items with the 4 trust dimensions.

#### Content Validation Framework

Both instruments underwent two rounds of content validation, assessing whether item content was relevant and appropriately framed, prior to psychometric testing: (1) dimensional content validation, to ensure item alignment with the correct trust dimension; and (2) contextual content validation, to ensure items were appropriate for pediatric brief-encounter settings.

Content validation required expert panels to rate item relevance on a 9-point Likert scale (1=“not at all relevant” to 9=“extremely relevant”) and to provide open feedback. A combined synthetic and human scale–content validity index (S-CVI) threshold of ≥0.80 was set for each instrument [29,30].

#### Human-Synthetic Collaborative Strategy

A simultaneous parallel evaluation design was performed, wherein human and synthetic panels completed assessments independently to ensure that human experts remained blind to synthetic outputs [31]. Both panels received identical instructions and contextual information. Human experts were recruited based on relevant professional or personal experience and completed a survey form to provide their ratings.

#### SIV Framework

The SIV process was governed by three human-centric principles: (1) complementary integration (LLMs augment rather than replace traditional human expert validation); (2) collaborative framework (human consensus serves as the primary reference standard, with synthetic processes serving a complementary role); and (3) human oversight (all SIV-suggested refinements require human expert confirmation and approval) [31].

#### Technical Infrastructure and Model Selection

All selected LLMs were accessed and operated within Perplexity Pro (Perplexity AI) workspaces, each provided with identical context, including the study protocol, the relevant trust instrument, and a relevant scoping review [14].

Three LLMs were selected based on observed reasoning abilities (September 2025) and accessibility within Perplexity AI. Claude Sonnet 4 was selected for its recognized lead in agentic reasoning and reduced tendency to rely on shortcuts or reasoning loopholes compared to earlier models [32]. GPT-5 was chosen for its advanced logical coherence, minimized hallucination rates, and slower, more deliberate reasoning process [33]. Grok 4 was selected for its demonstrated capacity for abstract reasoning, making it particularly relevant for evaluating conceptual domains such as the experience of trust in clinical settings [34].

#### Synthetic Expert Development

LLMs were prompted to embody several expert roles: pediatric emergentologist, pediatric general surgeon, qualitative researcher, emergency department (ED) patient family representative, and surgical outpatient patient family representative (5 for patient family instruments); and pediatric emergentologist, pediatric general surgeon, and quantitative researcher (3 for physician instruments). Prompt design followed best practices using a 4-part structure: role details, prompt, instructions, and parameters (Multimedia Appendix 1) [8].

Each synthetic expert involved a persona-model pairing where the pairing functioned as a single synthetic expert. Duplicate prompt runs were averaged as repeated measurements rather than as separate raters. A run was not included if it failed to provide a rating for every item. When a run failed, it was rerun to ensure 2 valid measurements per expert.

#### Two-Stage Content Validation Protocol

Content validation assessed the relevance of each instrument to measuring trust specifically within pediatric emergency and surgical care contexts. It was conducted in 2 stages. First, to evaluate the relevance of each item to its internal latent constructs, and, second, to assess the relevance of items to the external clinical context.

#### Dimensional Content Validation

Dimensional content validation involved assessing each item against its assigned trust dimension (dependability, caring, communication, and competence). A total of 3 human experts evaluated both types of instruments; in parallel, Claude Sonnet 4, GPT-5, and Grok 4 were persona-prompted to simulate patient-family and physician expert perspectives (see Multimedia Appendix 1 for detailed model parameters and prompt). Each item was displayed alongside its designated dimension, and experts rated using the 9-point Likert scale.

#### Contextual Content Validation

Items were further evaluated for their relevance to the broader pediatric emergency and surgical care context. Each item was rated on a 9-point Likert scale. The human panel included 7 experts for the patient-family instruments and 5 experts for the physician instruments. Welch *t* tests (1-tailed) were used to compare Item–Content Validity Index (I-CVI) rankings between each synthetic and human expert panel, identifying the optimal model for this stage.

#### Data Analysis - Content Validity Index

Item-level (I-CVI) and scale-level (S-CVI) content validity indices were calculated for both validation rounds. I-CVI was calculated by dividing the number of experts rating an item as relevant (7 out of 9 or higher) by all experts, calculated independently for the human and synthetic panels, and then combined. S-CVI was calculated as the average of I-CVIs (S-CVI/Ave) for each instrument, representing the proportion of content-valid items [4].

#### Data Analysis: Comparison of LLM to Human Ratings

Interrater reliability was assessed individually within human-only and synthetic-only panels for each round using fixed-marginal Fleiss κ, via Justus Randolph’s Fleiss κ calculator with a target of κ≥0.80 [35]. Items were categorized as either category 1 (ratings 0‐6) or category 2 (ratings 7‐9). Values were interpreted as κ<0.40 (“poor”), 0.40‐0.59 (“fair”), 0.60‐0.79 (“substantial”), and 0.80‐1.00 (“almost perfect agreement”) [36].

Percent agreement quantified the proportion of items classified equivalently as relevant or not relevant by both panels, per instrument and validation round. An item was classified as relevant for a given panel if at least two-thirds (66.7%) of that panel’s raters scored it ≥7. Items were flagged as a match if both panels’ classifications agreed.

To further characterize differences in rating tendencies between panels, mean relevance ratings on the original 9-point scale before binary categorization were calculated per expert across all items within a subsection and compared between the human-only and synthetic-only panels using Welch’s *t*-test consistent with the approach described for model selection in the contextual round.

#### Instrument Refinement

Final expert deliberation determined instrument modifications based on open-ended comments. Substantive synthetic panel comments were confirmed with the human panel or a survey methodologist before implementation.

### Phase 2: LLM-Assisted Instrument Metric Assessment

#### Overview

To evaluate the role of LLMs in quantitative tasks, instrument accessibility and semantic overlap were assessed using 3 parallel methods. The reference (“algorithmic”) method used Flesch-Kincaid indices for readability and embedding models with cosine similarity for semantic overlap. These metrics were then compared with 2 LLM-based approaches using Claude Sonnet 4.5 [37]: in the “LLM-instructed” condition, the model was explicitly directed to replicate the algorithmic procedure (ie, told to compute Flesch-Kincaid scores and cosine similarity using the same formulas as the reference method); in the “LLM-derived” condition, the model was asked to assess readability and semantic overlap directly, without being told which method or metric to use, allowing it to determine its own approach. Each LLM condition was run in triplicate, and the mean values were used for analysis.

#### Patient Family Readability Assessment

Flesch-Kincaid Reading Ease and Grade Level scores were calculated for the patient family instrument, including the demographic section (Multimedia Appendix 1) [38]. Readability was assessed using the 3 approaches described above, with the Readable software implementation of Flesch-Kincaid serving as the specified blueprint formula for use in the algorithmic method [39]. Claude Sonnet 4.5 was instructed to account for the regional context of Quebec, Canada. Targets were set to a Grade Level of ≤8 and a Reading Ease score of ≥60 [40]. All LLM threads were examined to identify semantic fragmentation, Python code generated, methods selected, and the decision rules guiding each computational pathway.

#### Semantic Overlap Assessment

To assess semantic overlap, pairwise cosine similarity was computed between all item pairs within each of the 4 instrument subsections, with pairs at a threshold of ≥0.90. As described in the *Phase 2: LLM-Assisted Instrument Metric Assessment* section, 3 methods were used, with OpenAI’s text-embedding-3-small model via a Python API serving as the reference algorithmic method [41].

Mean absolute error (MAE) was calculated to quantify the degree of deviation between the LLM-instructed and LLM-derived cosine similarity scores (each averaged across triplicate runs) and the corresponding algorithmic reference score, per item pair.

#### Evaluation of LLM-Assisted Performance and Prompt Validity

Each output across both tasks was evaluated to verify accurate computational pathways and results, and to characterize the reasoning underlying each approach. For the patient family readability analysis, all LLM threads were examined to identify the Python code generated, methods selected, and decision rules that determined each computational pathway and reasoning steps.

For the semantic overlap assessment, the researcher engaged LLMs in iterative discussion to derive reasoning pathways and document deviations from the algorithmic benchmark method. This iterative process yielded a prompt library for each LLM technique, which was then systematically reviewed by an adjudicator LLM to select optimal prompts based on reasoning quality and task alignment. The finalized prompts were subsequently tested to assess intertechnique performance variability and establish comparability with the algorithmic method. LLM discussions for each instrument and method type were transcribed, and thematic analysis was performed to identify key themes in LLM capacity to complete deterministic tasks.

### Ethical Considerations

The overall study falls under a larger umbrella study approved by the Research Ethics Board of McGill University (08/26/2025, 2025‐8251). All participants were provided with study information prior to participation, outlining the purpose and intended use of their responses. Consent was implied by the completion and return of the validation forms. No personally identifiable information was collected beyond participants’ relation to the study (eg, clinical role or family member status).

## Results

The trust instruments were evaluated both as a whole and by subsection, as appropriate, to account for differences between experience-based and criterion-based sections in terms of perspective and conceptual focus.

### Phase 1: Instrument Design and Content Validation

#### Experience-Based Trust (Subsection 1)

The WFTPS was adapted for pediatric emergency and surgical contexts, as outlined in Table 1 and further detailed in Multimedia Appendix 1, from which TRUST-E-F originated. The original 5-point Likert scale was modified to a 4-point scale (1=“No, not at all“ to 4=“Yes, definitely”) by removing the neutral midpoint. Verb tense was modified to emphasize acute rather than longitudinal care, “your doctor” was modified to “my child’s doctor” in all items, and selected items were reframed in first-person phrasing (“I felt”) to capture the patient family’s perspective; 8 of 10 original items best fitting the study context were selected, and 4 new items were added.

Wording was modified to align each item with a single dimension, per COSMIN (Consensus-Based Standards for the Selection of Health Measurement Instruments) guidelines [42]. The instrument was directly adapted to the physician perspective for TRUST-P-MD.

**Table 1.** Modifications of Wake Forest Trust in Physician Scale (WFTPS) for pediatric emergency and surgical contexts.

Original WFTPS item	Modifications
1 [Your doctor] will do whatever it takes to get you all the care you need.	Added “I felt” to highlight the patient-family reflection.
3 [Your doctor’s] medical skills are not as good as they should be.	Added “I felt” to capture the patient-family reflection, reworded positively.
4 [Your doctor] is extremely thorough and careful.	Added “appeared” to emphasize that this is how the patient family is feeling, as they may not know general standards of care for what they are seeking.
5 You completely trust [your doctor’s] decisions about which medical treatments are best for you.	Removed “completely” due to the brief nature of the pediatric setting and lack of prior relationship.
6 [Your doctor] is totally honest in telling you about all of the different treatment options available for your condition.	Removed “totally” to emphasize the fact that patient family may not know or have enough time to be aware of their child’s condition. Generalized from treatments to conditions to highlight the nature of surgical and emergency contexts.
7 [Your doctor] only thinks about what is best for you.	Added “I believe” to capture patient-family opinion.
8 Sometimes [your doctor] does not pay full attention to what you are trying to tell [him or her].	Added a layer of communication to both child and patient family, as well as reflection of their opinion.
10 All in all, you have complete trust in [your doctor].	“All in all” changed to “overall” to highlight entire consultation experience, given scale quantifies twice the experience of trust from the one visit.

#### Building Blocks of Trust (Subsection 2)

Review panel deliberations generated new items identified as essential building blocks of trust, with a 5-point Likert scale (1=“not at all important“ to 5=“extremely important”).

The distribution of items across trust dimensions for each instrument is shown in Table 2.

**Table 2.** Distribution of trust dimensions across patient family and physician instruments

Trust dimension	Patient family instrument		Physician instrument	
	TRUST-E-F^a^	TRUST-BB-F^b^	TRUST-P-MD^c^	TRUST-BB-MD^d^
Caring	2	4	2	4
Competence	3	2	2	2
Communication	3	2	2	2
Dependability	3	2	2	2
General	1	0	0	0
Total Items	12	10	8	10

a TRUST-E-F: Trust-Experience–Patient-Family.

b TRUST-BB-F: Trust-Building Blocks–Patient-Family.

c TRUST-P-MD: Trust-Perception–Physician.

d TRUST-BB-MD: Trust-Building Blocks–Physician.

#### Dimensional and Contextual Content Validation

For the dimensional round, 3 female human experts (ED patient family representative, pediatric emergentologist, and quantitative researcher) completed the pilot assessment for both patient family and physician instruments. This round took 2 weeks to complete, while synthetic experts provided evaluations within 2 hours. The distribution of synthetic experts can be found in Table 3. Synthetic patient family representatives were excluded from the physician instrument validation as they lack specialized clinical expertise, a gap that cannot be addressed in the absence of true lived experiences.

For the contextual round, 7 human experts were selected based on clinical or experience relevance to the instruments (1 pediatric general surgeon [male], 1 pediatric emergentologist [female], 1 pediatric nurse [female], 1 pediatric occupational therapist [female], 1 ED patient-family representative [female], and 2 doctoral students [females]). This group completed the patient family instrument assessment; five of the same experts (excluding 1 doctoral student and the pediatric nurse) completed the physician instrument assessment. This content validation round took the same amount of time as the dimensional round.

Based on the Welch *t* test (1-tailed) on the I-CVI scores of the dimensional content validation, Claude Sonnet 4 demonstrated fewer statistically significant differences from human expert panels (3 of 4 instruments) compared with GPT-5 or Grok 4 (2 of 4 instruments each) and was, therefore, selected for the contextual validation round.

S-CVI/Ave scores for the human, synthetic, and combined human-synthetic expert panels are shown in Table 4 (I-CVI scores of the panels; Multimedia Appendix 1). Each individual panel achieved S-CVI≥0.80 for both rounds and all instruments.

Fixed-marginal κ values were assessed at the subsection level for human-only and synthetic-only for the dimensional and contextual validation round as shown in Table 5.

**Table 3.** Distribution of synthetic expert personas by model and instrument across the dimensional validation stage. each synthetic expert averages ratings across duplicate prompt runs.

Instrument and model		Synthetic experts, n	Total, n
Patient family			15
Claude Sonnet 4		5	
GPT-5		5	
Grok 4		5	
Physician			9
Claude Sonnet 4		3	
GPT-5		3	
Grok 4		3	

**Table 4.** Scale-content validity index (S-CVI/Ave) for trust instruments of the human-only, synthetic-only, human-synthetic expert panel in dimensional and contextual content validation rounds.

Panel	S-CVI/Ave scores, mean (SD)			
	TRUST-E-F^a^	TRUST-BB-F^b^	TRUST-P-MD^c^	TRUST-BB-MD^d^
Dimensional validation				
Human panel	0.89 (0.16)	0.80 (0.16)	1.00 (0)	0.93 (0.13)
Synthetic panel	1.00 (0)	0.90 (0.17)	1.00 (0)	0.88 (0.26)
Human-synthetic panel	0.98 (0.03)	0.88 (0.16)	1.00 (0)	0.89 (0.19)
Contextual validation				
Human panel	0.89 (0.16)	0.93 (0.12)	0.95 (0.09)	0.92 (0.1)
Synthetic panel	0.97 (0.07)	1.00 (0)	1.00 (0)	1.00 (0)
Human-synthetic panel	0.92 (0.1)	0.96 (0.07)	0.97 (0.05)	0.95 (0.06)

a TRUST-E-F: Trust-Experience–Patient-Family.

b TRUST-BB-F: Trust-Building Blocks–Patient-Family.

c TRUST-P-MD: Trust-Perception–Physician.

d TRUST-BB-MD: Trust-Building Blocks–Physician.

**Table 5.** Fleiss κ values of the trust instrument subsections across dimensional and contextual content validation rounds.

Subsection	Interrater agreement κ (95% CI)	
	Human panel	Synthetic panel
Dimensional validation		
TRUST-E-F^a^	0.56 (0.18 to 0.93)	1.00 (1.00-1.00)
TRUST-BB-F^b^	0.20 (–0.23 to 0.63)	0.74 (0.47-1.00)
TRUST-P-MD^c^	1.00 (1.00 to 1.00)	1.00 (1.00-1.00)
TRUST-BB-MD^d^	0.73 (0.38 to 1.00)	0.79 (0.51-1.00)
Contextual validation		
TRUST-E-F	0.67 (0.41 to 0.92)	0.87 (0.69-1.00)
TRUST-BB-F	0.75 (0.50 to 1.00)	1.00 (1.00-1.00)
TRUST-P-MD	0.80 (0.54 to 1.00)	1.00 (1.00-1.00)
TRUST-BB-MD	0.68 (0.42 to 0.94)	1.00 (1.00-1.00)

a TRUST-E-F: Trust-Experience–Patient-Family.

b TRUST-BB-F: Trust-Building Blocks–Patient-Family.

c TRUST-P-MD: Trust-Perception–Physician.

d TRUST-BB-MD: Trust-Building Blocks–Physician.

In the human-only panel subsections, TRUST-E-F and TRUST-BB-F did not meet the κ≥0.80 threshold in both rounds (TRUST-E-F: dimensional κ=0.56, 95% CI 0.18-0.93 and contextual κ=0.67, 95% CI 0.41-0.92; TRUST-BB-F: dimensional κ=0.20, 95% CI –0.23 to 0.63 and contextual κ=0.75, 95% CI 0.50-1.00). Despite this, S-CVI met the threshold for both subsections and rounds, and mean raw relevance ratings did not differ significantly between human and synthetic panels in the dimensional round for TRUST-E-F and TRUST-BB-F (*P*=.21 and *P*=.65, respectively).

Percent agreement between human and synthetic panel classifications was calculated at the item level. In the dimensional validation round, agreement was 90.9% for the patient family instrument and 88.9% for the physician instrument. In the contextual validation round, agreement was 90.9% for the patient family instrument and 100% for the physician instrument; the lowest agreement observed at the subsection level was 80% in subsections TRUST-BB-F and TRUST-BB-MD in the dimensional round (Multimedia Appendix 1).

Mean raw relevance ratings (1‐9 scale) were higher among synthetic-only panels compared to human-only panels in 6 of the 8 subsections, where the mean difference for TRUST-E-F in the contextual round was statistically significant (mean difference=0.58; *P*=.039); the remaining comparisons did not reach significance (Multimedia Appendix 1). A pattern was observed for both rounds in which synthetic experts tended to rate the items as more relevant (“synthetic optimism”), whereas human experts tended to rate them more critically (“human conservatism”).

#### Instrument Refinement

Refinements included adjusting wording for better contextual fit, removing double-barreled items where a single item covered 2 or more concepts, and bolding key aspects of the item to draw respondents’ attention to the main points (Multimedia Appendix 1) [43,44]. One TRUST-E-F item (“I felt my child’s doctor was honest with me about my child’s condition”) was removed based on human expert majority consensus (4 of 7), despite favorable I-CVI scores (dimensional: 0.97, contextual: 0.94), because of redundancy with item 3 (“My child’s doctor seemed transparent when discussing my child’s condition, even when the news was challenging”), which received a perfect I-CVI in both rounds.

### Phase 2: LLM-Assisted Readability Evaluation

Phase 2 was conducted following completion of phase 1 content validation and instrument refinement; the refined patient family instrument underwent Flesch-Kincaid assessments, and both refined instruments underwent cosine similarity analyses.

#### Patient Family Readability Assessment

Flesch-Kincaid readability scores across the patient family instrument and its subsections are shown in Table 6. The algorithmic method meets the target threshold for Grade Level (6.9) but falls marginally below the target Reading Ease score (58.2).

Analysis of LLM-generated code confirmed appropriate computation of Flesch-Kincaid in all runs where follow-up queries to address discrepancies identified syllable, sentence, and word counting rules as sources of deviation; for instance, certain runs used manual counting while others used a library to determine syllable counts. Further analysis of the coding logic confirmed that no glitches or incorrect routes were taken (eg, incorrect or alternative formula usage or variable assignments). The average of the 3 methods for the overall instrument met target thresholds (Grade Level 8.1 SD 1.1; Reading Ease 60.1 SD 5.6).

**Table 6.** Flesch-Kincaid Reading Ease and Grade Level scores for the patient family instrument across 3 methods.

Section	Technique					
	Algorithmic, mean		LLM^a^-derived, mean (SD)		LLM-instructed, mean (SD)	
	Grade Level	Reading Ease	Grade Level	Reading Ease	Grade Level	Reading Ease
Overall	6.9	58.2	8.9 (0.7)	66.4 (17.8)	8.4 (1.1)	55.8 (5.5)
Demographics	7.6	46.8	6.9 (1.0)	62.1 (32.3)	8.1	47.0 (15.6)
TRUST-E-F^b^	7.6	69.5	8.0 (0.2)	69.9 (4.4)	7.9	67.1 (1.2)
TRUST-BB-F^c^	10.2	47.1	9.9 (0.5)	61.4 (10.8)	9.5	53.2 (1.37)

a LLM: large language model.

b TRUST-E-F: Trust-Experience–Patient-Family.

c TRUST-BB-F: Trust-Building Blocks–Patient-Family.

#### Semantic Overlap Assessment

Cosine similarity scores for the trust instrument subsections can be visualized in Figure 2. The highest semantic overlap based on the algorithmic method for each instrument is listed in Table 7. The highest cosine similarity scores for item pairs identified by the mean across the 3 approaches (algorithmic, LLM-derived, and LLM-instructed) were between TRUST-E-F items 5 and 7 (0.61, SD 0.41), TRUST-BB-F items 7 and 8 (0.69, SD 0.24), TRUST-P-MD items 1 and 2 (0.53, SD 0.28), and TRUST-BB-MD items 7 and 8 (0.63, SD 0.27). Item-to-item comparison values for all pairs are provided in Multimedia Appendix 1.

The MAE for the LLM-derived technique was TRUST-E-F of 0.12, TRUST-BB-F of 0.15, TRUST-P-MD of 0.17, and TRUST-BB-MD of 0.13. For the LLM-instructed technique, the MAE was TRUST-E-F of 0.46, TRUST-BB-F of 0.39, TRUST-P-MD of 0.42, and derived TRUST-BB-MD of 0.40.

**Figure 2.**
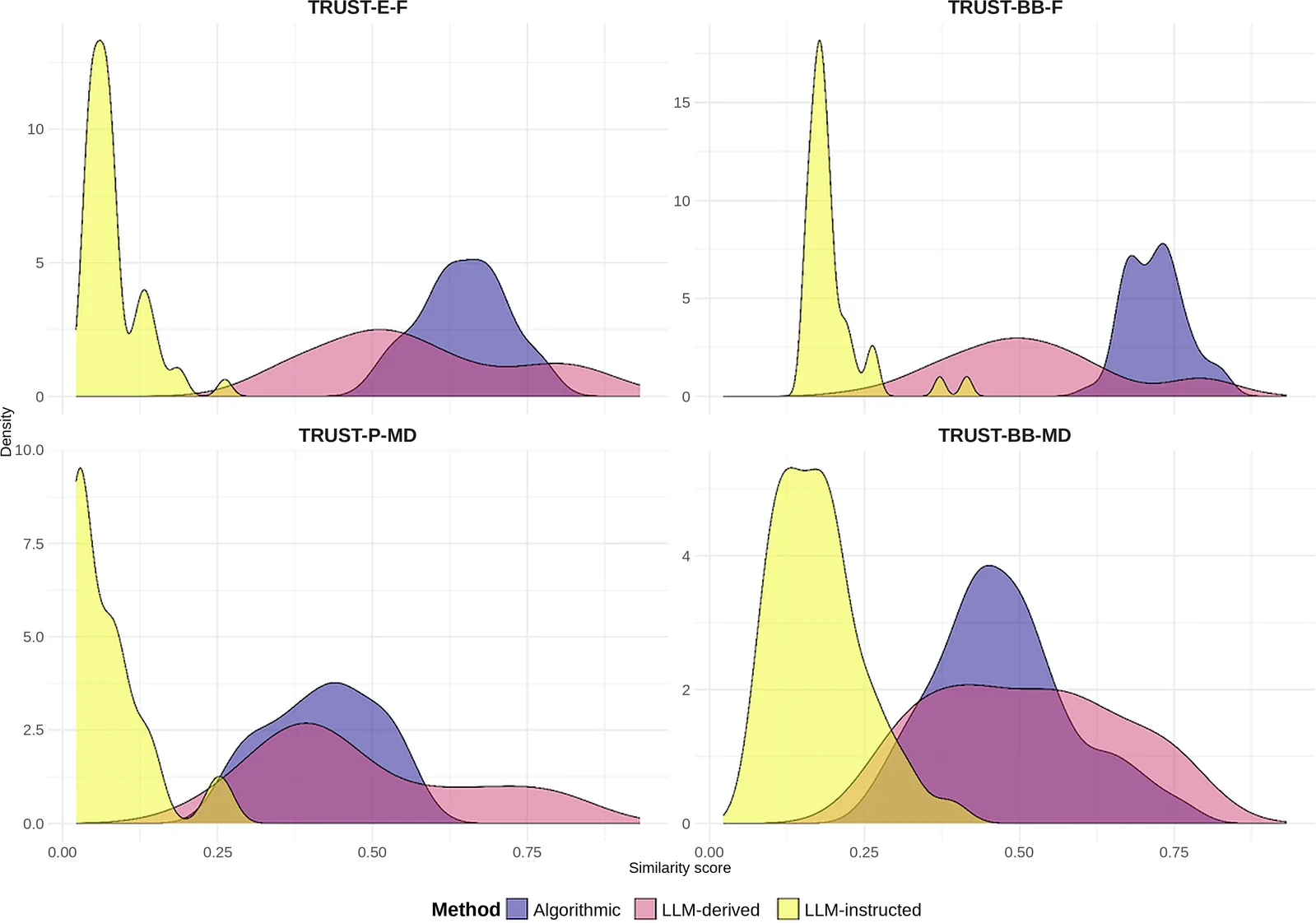
Cosine similarity score distributions across the 3 methods (algorithmic, LLM-derived, and LLM-instructed) for each instrument. TRUST-E-F: Trust-Experience–Patient-Family; TRUST-BB-F:Trust-Building Blocks–Patient-Family; TRUST-P-MD: Trust-Perception–Physician; TRUST-BB-MD:Trust-Building Blocks–Physician.

**Table 7.** Cosine similarity scores of the highest item pair of the trust instruments using the algorithmic method.

Instrument (Section)	Item text	Similarity score
Patient Family (TRUST-E-F^a^)	I felt as though my child’s doctor focused on what was best for my child’s medical needs. I felt my child’s doctor did whatever it took to get my child all the care they needed.	0.78
Patient Family (TRUST-BB-F^b^)	My child’s doctor knowledgeably addressing my concerns about my child’s treatment. My child’s doctor managing my child’s treatment effectively.	0.83
Physician (TRUST-P-MD^c^)	My ability to explain things clearly. My reliability in following through on what I say.	0.55
Physician (TRUST-BB-MD^d^)	Treating patient families with respect. Ensuring patient families feel understood in their concerns.	0.76

a TRUST-E-F: Trust-Experience–Patient-Family.

b TRUST-BB-F: Trust-Building Blocks–Patient-Family.

c TRUST-P-MD: Trust-Perception–Physician.

d TRUST-BB-MD: Trust-Building Blocks–Physician.

#### Evaluation of LLM-Assisted Performance and Prompt Validity

The adjudicator selections for the LLM-derived and LLM-instructed methods are visualized in Multimedia Appendix 1. Based on iterative discussions with LLMs and their inability to execute appropriate methods with the selected prompts, the final cosine similarity scores relied solely on the algorithmic scores.

Following thematic analysis of the 8 LLM transcripts, 4 key themes were identified as factors contributing to inappropriate execution: tool constraints pushing incorrect approximations, LLMs being probabilistic rather than deterministic, helpfulness being prioritized over accuracy, and an inability to maintain consistency, as highlighted in Multimedia Appendix 1.

## Discussion

### Principal Findings

We content-validated 2 pediatric trust instruments, one for patient families (TRUST-E-F and TRUST-BB-F) and another for physicians (TRUST-P-MD and TRUST-BB-MD), using parallel human and synthetic expert panels through a new approach termed SIV. This serves as a crucial preliminary step toward further instrument validation with patient families.

All instruments met or exceeded the established S-CVI/Ave threshold of 0.80 across human and synthetic panels. These results surpass content validity literature benchmarks, suggesting that the instruments appropriately measure trust dimensions while maintaining contextual relevance to pediatric emergency and surgical care settings [29]. Percent agreement between human and synthetic panels was high across both validation rounds (88.9%‐100%), indicating strong alignment in relevance classifications despite the panels’ structurally distinct compositions. Within-panel reliability, however, varied by round: in the contextual round, where human panels were larger (n=5‐7), κ values remained substantial to almost perfect across subsections (κ=0.67‐0.80); in the dimensional round, comprising only 3 human experts, κ values were considerably less stable, ranging from poor (TRUST-BB-F, κ=0.20) to almost perfect (TRUST-P-MD, κ=1.00), despite still achieving target S-CVI scores. Panel size alone does not fully account for this pattern, however: in the physician instrument’s contextual round, the synthetic panel (n=3) was smaller than the human panel (n=5) yet achieved perfect agreement (κ=1.00), while the human dimensional panel showed instability at the same n value of 3. It is possible that κ values may have sensitivity to small samples that manifests differently across panel types, with synthetic panels retaining internal consistency at low n and human panels showing greater categorical variability at the same size. This may reflect a difference in panel composition rather than sample size, as human panels comprised individually distinct experts with varied backgrounds and clinical roles, whereas synthetic panels, though persona-differentiated, remained anchored to a shared underlying model baseline, which may narrow rating variability even at low n. Small panel size, therefore, appears to interact differently with human versus synthetic rater composition, rather than acting as a uniform statistical limitation across panel types.

Traditional content validation typically requires panels of 5 to 10 experts over extended timelines [29]. Our SIV approach completed both validation rounds within 4 weeks with a minimum of 8 experts evaluating each instrument, while maintaining robust validity metrics. That speed can address a fundamental barrier in specialized populations where expert availability is limited, such as in pediatrics [45,46]. These findings align with growing evidence suggesting that LLMs can approximate human evaluator agreement in health care settings while substantially reducing the time and resources required for validation. For example, GPT-o3-mini achieved an intraclass correlation coefficient of 0.82 with human expert evaluators when evaluating electronic health record quality [47], while GPT-4o has shown validity as a psychological text classifier [7]. Yet most AI applications have focused on evaluating completed instruments rather than developing new ones, making this study among the first to demonstrate LLMs’ potential in preliminary content validation [31].

Across repeated runs, LLMs consistently adopted their assigned perspective, providing ratings and justifications grounded in personal or knowledge-based reasoning, with subtle variations across reruns enriching the comparability of the dataset. For example, a synthetic patient family representative (Claude Sonnet 4, dimensional round) noted: *“*As a parent, my confidence in the doctor’s ability to handle my child’s medical issues is fundamental to trust.” These outputs demonstrate the model’s capacity to synthetically emulate expert reasoning, meaningfully augmenting the content validation process through diverse yet contextually relevant insights.

However, this capacity for emulation does not equate to the accountability or lived experience inherent to human judgment, and synthetic personas were not intended to substitute for either. The SIV framework’s guiding principles instead ensured that no synthetic-suggested refinement was implemented without human expert confirmation. The same reasoning underlies our exclusion of synthetic patient-family personas from physician instrument validation: while synthetic outputs may complement human judgment by surfacing patterns for further evaluation, they cannot substitute for the lived clinical or patient-family experience that these tasks require.

Of note, synthetic experts demonstrated a tendency to rate items more positively, while human experts provided more critical evaluations. This complementary dynamic, which we term “synthetic optimism” and “human conservatism,” proved advantageous; synthetic optimism ensured efficient consensus-building and progression through validation stages, while human scrutiny identified opportunities for refinement [48]. This tendency was most consistent throughout patient-family instruments, where synthetic ratings exceeded human ratings in both rounds. Human critique also drove the removal of one TRUST-E-F item, further evidence of such a pattern. We, therefore, argue that human and synthetic experts may function synergistically, rather than as interchangeable panels, consistent with collaborative AI frameworks where differing capabilities are intended to augment rather than replace one another [25]. Of note, only 1 of 8 subsection comparisons reached significance; therefore, this should be interpreted as a pattern worth further investigation rather than a confirmed phenomenon.

The patient-family instrument’s average readability score across all 3 methods met predefined thresholds (Grade Level 8.1, SD 1.1; Reading Ease: 60.1, SD 5.6). The algorithmic reference method alone fell slightly short of the Reading Ease target of 58.2, though it met the Grade Level target (6.9). These deviations primarily reflected differences in computational approaches (ie, syllable, sentence, and word counts) rather than substantive performance gaps, with both LLM-derived and LLM-instructed methods diverging modestly from the algorithmic result as a function of these implementation differences, not a difference in the underlying technique. This pattern is consistent with known limitations of automated readability tools: even calculators nominally applying the same formula have been shown to diverge by as much as 12.9 grade levels on identical text due to differences in sentence- and syllable-counting conventions [49]. Rather than treating this as a limitation unique to LLM-based computation, we interpret our 3-condition average as a way of reducing sensitivity to any single implementation’s counting idiosyncrasies, consistent with this broader, well-documented source of variability in readability measurement [49,50].

Cosine similarity and readability outcomes were ultimately handled differently based on LLMs’ differential ability to execute simple versus complex computational tasks. Semantic overlap analysis revealed no within-instrument redundancy, as reflected in Table 7. TRUST-BB-F items 1‐2 showed the highest cosine similarity scores (0.83; algorithmic method), likely reflecting their shared competence construct. LLM approaches underperformed due to their dependence on word frequency counts rather than on complex semantic algorithms, exposing their limitations for tasks requiring deterministic outputs [51].

LLMs excelled at human-like content validation reasoning but struggled with reproducible complex computation, evidenced by cosine similarity MAE values of 0.14 (LLM-derived) and 0.42 (LLM-instructed) relative to the algorithmic reference. These findings may reflect that in this context, LLMs are probabilistic models not yet suited for complex, stepwise algorithmic pipelines, deviating from specified methods despite clear prompting. This can be attributed to tool access limitations and training that rewards confident yet incorrect responses over honest acknowledgment of capability boundaries [52]. Thematic analysis of the LLM conversations identified 4 mechanisms underlying these failures: deeper governing rules that overrode explicit methodological prompts; episodes of reasoning dishonesty, wherein frequency-based outputs were mislabeled as “semantic embeddings” or “cosine similarity”; insufficient technical capacity to access the exact embedding and readability pipelines used as ground truth; and a tendency to favor computational shortcuts over more rigorous but slower methods. Together, these behaviors indicate that using LLMs as independent coquantitative researchers is not currently recommended, as direct human oversight, while essential for result determination, is impractical for complex pipelines and would undermine the central SIV objective of improving the feasibility of instrument development [53,54].

At this stage, the candidate SIV approach would integrate stochastic LLMs for qualitative expert simulation, deterministic models for quantitative tasks, and human governance to ensure rigor. This could be strategically implemented through an AI coresearcher capable of receiving tasks, classifying their nature, and delegating them to appropriate models. This will ensure that AI is used intentionally, producing reliable and reproducible metrics, while leveraging LLMs’ creative reasoning to match capabilities to task requirements.

The SIV framework has the potential for evaluation in subsequent validation rounds, beginning with cognitive interviewing [1,30,55]. Once the potential for SIV to function within the entire instrument validation process is established, it should be evaluated for applicability to broader health care contexts such as oncology, mental health, and quality of care measurement [56]. However, adaptation would require context-specific persona development and equally rigorous validation of synthetic response patterns against real-world populations [57]. Standardized SIV guidelines should specify deep learning model selection criteria, human-synthetic panel composition, and quality assessment protocols. As AI becomes more extensively involved in instrument validation, transparency and accountability must be ensured through the integration of established frameworks such as FUTURE-AI [58].

### Reflexivity

The lead author conducted this work as a graduate student at a well-positioned Canadian academic institution with ready access to commercial LLM infrastructure and established clinical research networks that are not uniformly available to researchers in lower-resource settings. While the broader team brings expertise in psychometric design and pediatric surgical care, the primary methodological innovation was led by a researcher trained in AI and health research methodology, whose positionality may have shaped key design decisions. The 4 trust dimensions identified through this process may reflect a conceptualization of the patient-physician relationship shaped by Western academic and clinical discourse, which may not fully capture how trust is experienced across the cultural and linguistic diversity of families in pediatric surgical settings. If instruments rely solely on LLM validation, the perspectives of the patient families that these tools are designed to serve—particularly those from equity-seeking communities underrepresented in LLM training data risk systematic exclusion from the development process. The SIV framework was designed as a complementary step to reduce validation burden, not to substitute lived experience expertise; however, the authors acknowledge the risk that downstream adoption could erode community engagement in instrument development if applied without the guiding principles central to this framework.

### Limitations

Our study has several limitations. First, the rapid advancement of LLM capabilities means that the models evaluated in this study (up to September 2025) may not reflect frontier model performance by the time of publication. Second, this study focused exclusively on the preliminary validation of the instruments; the applicability of SIV to subsequent instrument analysis such as cognitive interviewing and pilot testing remains to be empirically established. Third, the study was conducted within a single health care context, which may limit generalizability and will require further evaluation to establish the robustness of SIV across diverse settings and constructs. In addition, the human panel in the dimensional round (n=3) and the physician synthetic personas were smaller than the typically recommended size for content validation [29]. Future SIV work should ensure adequately sized panels throughout. Relatedly, while persona-differentiated synthetic experts address pseudoreplication, they remain anchored to a shared underlying model in a way independent human experts are not; each persona varies the reasoning frame applied to a task, but not the model producing that reasoning. This may affect the independence of synthetic panel members relative to human ones. As a result, the Fleiss κ and content validity index calculations should be interpreted as descriptive indices of within-panel and cross-panel agreement rather than as formally independent psychometric estimates for the synthetic panel. Finally, cosine similarity results prompted no instrument modifications; future comparison with cognitive interviewing outcomes will guide potential item revision or elimination.

### Conclusions

This study demonstrates the feasibility of the SIV approach, using persona-prompted LLMs to augment the initial steps of health care instrument development. While results support preliminary content validity, full psychometric testing with patient families and physicians is required before these instruments can be considered validated for clinical use. The pediatric trust instruments successfully achieved content validation thresholds with substantial human-synthetic expert agreement, enabling their progression to further psychometric testing. Beyond validating these specific instruments, this work offers a preliminary methodological blueprint for accelerating instrument development while delineating appropriate use cases for LLM integration [24]. Future iterations could use AI agents to route tasks strategically to either deterministic models for quantitative precision or agentic LLMs for creative, human-like reasoning that benefits from stochastic variation.

The SIV approach offers a way to address barriers that have historically constrained health care instrument development [59,60]. As AI continues to advance, SIV could evolve into a rigorous, scalable framework for developing health care instruments that strengthen evidence-based care across clinical settings.

## Supplementary material

10.2196/96901 Multimedia Appendix 1 File containing additional methodological detail and supporting data, including: synthetic expert persona set up and prompts (S1), patient-family demographic section (S2), Wake Forest Trust in Physician Scale (WFTPS) adaptation and item origin documentation (S3), item–content validity index scores (S4), percent agreement of human-synthetic panels (S5), mean raw relevance scores across panels (S6), instrument refinement log (S7), pairwise cosine similarity values (S8), adjudicator large language model (LLM) prompt library selections (S9), and transcript excerpts for the thematic analysis (S10).

## Abbreviations

ED: emergency department
I-CVI: item–content validity index
LLM: large language model
MAE: mean absolute error
PEDI-TiPS: Pediatric Trust in Physician Scale
S-CVI: scale–content validity index
SIV: Synthetic Instrument Validation
TRUST-BB-F: Trust-Building Blocks–Patient-Family
TRUST-BB-MD: Trust-Building Blocks–Physician
TRUST-E-F: Trust-Experience–Patient-Family
TRUST-P-MD: Trust-Perspective–Physician
WFTPS: Wake Forest Trust in Physician Scale
